# 24-h bronchodilation and inspiratory capacity improvements with glycopyrrolate/formoterol fumarate via co-suspension delivery technology in COPD

**DOI:** 10.1186/s12931-017-0636-4

**Published:** 2017-08-18

**Authors:** Colin Reisner, Gregory Gottschlich, Faisal Fakih, Andras Koser, James Krainson, Luis Delacruz, Samir Arora, Gregory Feldman, Krishna Pudi, Shahid Siddiqui, Chad Orevillo, Andrea Maes, Earl St. Rose, Ubaldo Martin

**Affiliations:** 1 AstraZeneca, Inc., Gaithersburg, MD USA; 2 Pearl Therapeutics, Inc., 280 Headquarters Plaza, East Tower, Morristown, NJ 07960 USA; 3 New Horizons Clinical Research, Cincinnati, OH USA; 4 Florida Pulmonary Research Institute, Winter Park, FL USA; 5 Palmetto Medical Research Associates, Easley, SC USA; 6 Clinical Trials of Florida, Miami, FL USA; 7 Greenville Pharmaceutical Research, Greenville, SC USA; 8 Aventiv Research, Columbus, OH USA; 9 S. Carolina Pharmaceutical Research, Spartanburg, SC USA; 10 Upstate Pharmaceutical Research, Greenville, SC USA; 11 Former employee of Pearl Therapeutics, Inc., Morristown, NJ USA

**Keywords:** COPD, Muscarinic antagonists, β_2_-agonist, Co-suspension delivery technology, Metered dose inhaler, Smoking, Chronic bronchitis, Emphysema, Bronchodilator

## Abstract

**Background:**

Symptoms of chronic obstructive pulmonary disease may vary throughout the day and it is important that therapeutic approaches provide 24-h symptom control. We report the results of two phase IIIb crossover studies, PT003011 and PT003012, investigating the 24-h lung function profile of GFF MDI (glycopyrrolate/formoterol fumarate 18/9.6 μg delivered using innovative co-suspension delivery technology) administered twice daily.

**Methods:**

Patients with moderate-to-very severe chronic obstructive pulmonary disease received 4 weeks’ treatment with each of GFF MDI, placebo MDI, and open-label tiotropium (PT003011 only). Lung function was assessed over 24 h on day 29 of each treatment period. The primary outcome was forced expiratory volume in 1 second area under the curve from 0 to 24 h (FEV_1_AUC_0–24_). Other outcomes included change from baseline in average daily rescue medication use over the treatment period. In addition, we conducted a post-hoc analysis of data pooled from both studies to further characterize the effect of GFF MDI on inspiratory capacity.

**Results:**

GFF MDI treatment significantly increased FEV_1_AUC_0–24_ versus placebo in studies PT003011 (*n* = 75) and PT003012 (*n* = 35) on day 29 (both studies *p* < 0.0001), with similar improvements in FEV_1_AUC versus placebo for hours 0–12 and 12–24. In PT003011, improvements with GFF MDI versus tiotropium in FEV_1_AUC were greater during hours 12–24 compared to 0–12 h. GFF MDI treatment also resulted in a significant reduction in rescue medication use versus placebo (−0.84 [*p*<0.0001] and −1.11 [*p*=0.0054] puffs/day in PT003011 and PT003012, respectively), and versus tiotropium in PT003011 (−0.44 [*p*=0.017] puffs/day). A post-hoc pooled analysis showed patients treated with GFF MDI were more likely to achieve a >15% increase from baseline in inspiratory capacity than patients treated with placebo or tiotropium (72.1%, 19.0% and 47.0% of patients, respectively after the evening dose on day 29). There were no significant safety/tolerability findings.

**Conclusions:**

GFF MDI significantly improved 24-h lung function versus placebo in patients with moderate-to-very severe chronic obstructive pulmonary disease, with similar benefits in the second 12-h period compared to the first, supporting twice-daily dosing of GFF MDI.

**Trial registration:**

Pearl Therapeutics, Inc.; www.clinicaltrials.gov; NCT02347072 and NCT02347085. Registered 21 January 2015.

## Background

Long-acting bronchodilators have become a key treatment choice for the management of chronic obstructive pulmonary disease (COPD) [1]. High-quality evidence from multiple clinical trials suggests that combination treatment with a long-acting muscarinic antagonist (LAMA) and a long-acting β_2_-agonist (LABA) reduces symptoms compared to LAMA or LABA monotherapy [2]. COPD symptoms tend to vary throughout the day [3–5], and despite treatment, many patients with COPD experience symptoms throughout the whole 24-h day, including night-time and early morning symptoms [6, 7]. Patients who experience symptoms during any part of the 24-h day have significantly worse outcomes across patient-reported measures (including health status, sleep quality, anxiety, and depression) compared with asymptomatic patients [7], highlighting the need for therapeutic approaches that provide 24-h symptom control.

GFF MDI is a twice daily (BID) LAMA/LABA fixed-dose combination (FDC) of glycopyrrolate/formoterol fumarate 18/9.6 μg (equivalent to glycopyrronium/formoterol fumarate dihydrate 14.4/10 μg) delivered by metered dose inhaler (MDI) using innovative co-suspension delivery technology. The co-suspension delivery technology provides a strong, non-specific association between drug crystals and porous particles, allowing uniform dose delivery [8]. The efficacy of GFF MDI in improving lung function over 24 weeks versus its monocomponent MDIs was previously evaluated in two pivotal phase III clinical studies (PINNACLE-1 [NCT01854645] and PINNACLE-2 [NCT01854658]) [9], which led to approval of GFF MDI (Bevespi Aerosphere™) for the long-term, maintenance treatment of airflow obstruction in patients with COPD in the USA [10]. A 28-week safety extension of these studies (PINNACLE-3 [NCT01970878]), comparing GFF MDI to its monocomponent MDIs and open-label tiotropium, provided further evidence of a favorable benefit-risk profile [11]*.*

Here, we present the results from two phase IIIb studies, PT003011 (NCT02347072) and PT003012 (NCT02347085), characterizing the 24-h lung-function profile of GFF MDI BID relative to placebo MDI BID in patients with moderate-to-very severe COPD. PT003011 also included open-label tiotropium 5 μg (Spiriva® Respimat®), administered once daily (QD) using a Soft Mist™ Inhaler (SMI) as an active comparator. In addition, we performed a post-hoc analysis of data pooled from both studies to further evaluate the effect of GFF MDI on inspiratory capacity.

## Methods

### Study design and treatment

PT003011 and PT003012 were crossover, multicenter, randomized, double-blind studies conducted in the USA. The studies had similar designs, with the exception of the inclusion of an open-label tiotropium SMI arm in PT003011. In study PT003011, patients were randomly assigned to one of six treatment sequences, each comprising three 4-week periods of treatment with the following in a crossover fashion: GFF MDI 18/9.6 μg BID; placebo MDI BID; and open-label tiotropium SMI 5 μg QD. In study PT003012, patients were randomly assigned to one of two treatment sequences, comprising two 4-week periods with GFF MDI and placebo MDI treatment administered in a crossover fashion.

Patients were required to discontinue any previous COPD medications during both studies, and were provided with Atrovent® HFA (ipratropium bromide inhalation aerosol, four times daily) and rescue Ventolin® HFA (albuterol sulfate inhalation aerosol, up to four times daily as required) to control symptoms. Use of albuterol sulfate was permitted during the treatment and washout periods as rescue medication. However, ipratropium bromide was only for use during the washout periods and was replaced with study drug during treatment periods. Patients previously using an inhaled corticosteroid as part of an FDC were switched to an equivalent inhaled corticosteroid monotherapy with fluticasone, mometasone, or budesonide.

These studies were conducted in accordance with Good Clinical Practice guidelines including the International Council on Harmonisation, the US Code of Federal Regulations, and the Declaration of Helsinki. All patients provided written informed consent prior to the performance of any screening evaluations.

### Patients

Patients eligible for inclusion in PT003011 and PT003012 were 40 to 80 years of age; had moderate-to-very severe COPD (as defined by the American Thoracic Society/European Respiratory Society criteria) [12]; and were current or ex-smokers with a history of ≥10 pack-years. Patients who had been hospitalized due to COPD in the past 3 months, had poorly controlled COPD, had changed their smoking status during screening, or required long-term oxygen therapy for >12 h per day were excluded. Both studies allowed patients to withdraw at any point, and investigators could request withdrawal of a patient if they met any of the pre-specified discontinuation criteria.

### Objectives and endpoints

The objective of these studies was to determine the 24-h efficacy profile of GFF MDI relative to placebo MDI in patients with moderate-to-very severe COPD following chronic dosing (over a 4-week period), with the additional objective of characterizing this profile relative to open-label tiotropium SMI in PT003011.

The primary efficacy outcome for both studies was forced expiratory volume in 1 s (FEV_1_) area under the curve from 0 to 24 h (AUC_0–24_) on day 29. The 24-h efficacy profile of GFF MDI was further characterized using secondary efficacy outcomes, including FEV_1_ AUC_12–24_ and FEV_1_ AUC_0–12_ on day 29; peak change from baseline in FEV_1_ following evening and morning doses on day 29; change from baseline in morning pre-dose trough FEV_1_ on days 29 and 30; and peak change from baseline in inspiratory capacity following evening and morning doses on day 29. Other endpoints included change from baseline in average daily rescue medication use over the treatment period.

### Assessments

Study visits were scheduled on day 1 and day 29 of each treatment period. On day 29, spirometry was assessed 60 and 30 min prior to drug administration; 15 and 30 min post-dose; and at 1, 2, 4, 8, 11.5, 12, 12.25, 12.5, 13, 14, 16, 22, 23.5, and 24 h post-dosing. Investigators assessed continued eligibility for study participation at each visit. Safety data, including adverse events (AEs), vital sign measurements, electrocardiograms, and clinical laboratory testing, were collected throughout the study.

### Statistical analysis

Assuming a patient dropout rate of 20%, a sample size of 80 randomized patients was estimated to provide 99% power to detect a difference of 200 mL in the primary endpoint for GFF MDI versus placebo MDI with a two-sided alpha level of 0.05; and also to provide 90% power to demonstrate a difference of 75 mL in the primary endpoint between GFF MDI and open-label tiotropium SMI in PT003011. For PT003012, the number of patients necessary to detect a difference of 200 mL in the primary endpoint for GFF MDI versus placebo MDI with a two-sided alpha level of 0.05 was 40.

The primary analyses were conducted using the modified intent-to-treat population (mITT), the subset of the intent-to-treat population that included all patients who received treatment and provided post-treatment efficacy data from at least two treatment periods. The primary endpoint was analyzed using a mixed model, with baseline FEV_1_ as a continuous covariate, and period and treatment as unordered categorical covariates. Subject was included as a random effect to model correlation within subject across the study. Secondary endpoints based on FEV_1_, inspiratory capacity, or rescue medication use were analyzed using the same model, with the only difference being that the baseline value was specific to the endpoint.

#### Post-hoc analysis of pooled data

The effect of GFF MDI on inspiratory capacity was further evaluated in a post-hoc analysis of pooled data from both studies (mITT population). Inspiratory capacity responder analyses, using various thresholds, were conducted using a logistic regression model with covariates for study, baseline inspiratory capacity, treatments nested within study, and period nested within study. Point estimates with 95% confidence intervals were produced for each treatment difference, with no adjustments made for multiplicity.

## Results

### Patients

**Fig. 1:**
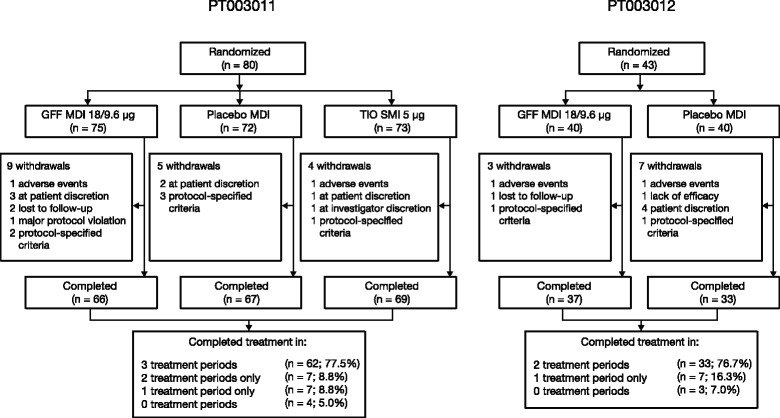
Patient disposition. GFF, glycopyrrolate/formoterol fumarate; MDI, metered dose inhaler; SMI, Soft Mist™ inhaler; TIO, open-label tiotropium

In study PT003011, 80 patients were randomized to one of six treatment sequences; whilst in PT003012, 43 patients were randomized to one of two treatment sequences (Fig. 1). Overall, 75 patients were included in the mITT population in PT003011 and 35 patients in PT003012. Patient demographics were generally similar across both studies and treatment groups (Table 1). The higher proportion of female patients in study PT003011 compared to PT003012 (64.0% vs 42.9%) was not considered to be clinically relevant. Current smoking status and duration of COPD were similar across groups in both of the studies.

**Table 1:** Patient demographics and baseline characteristics (mITT population)

	PT003011			PT003012	
	GFF MDI 18/9.6 μg (*n* = 73)	Placebo MDI (*n* = 69)	TIO SMI 5 μg (*n* = 73)	GFF MDI 18/9.6 μg (*n* = 35)	Placebo MDI (*n* = 35)
Mean age, years (SD)	61.9 (9.1)	61.7 (9.1)	61.9 (8.9)	61.3 (9.2)	61.3 (9.2)
Male, *n* (%)	26 (35.6)	24 (34.8)	27 (37.0)	20 (57.1)	20 (57.1)
White, *n* (%)	66 (90.4)	63 (91.3)	66 (90.4)	27 (77.1)	27 (77.1)
Current smokers, *n* (%)	45 (61.6)	43 (62.3)	45 (61.6)	20 (57.1)	20 (57.1)
Mean smoking history, pack-years (SD)	56.4 (29.2)	57.6 (29.3)	55.9 (29.3)	49.0 (25.2)	49.0 (25.2)
Use of ICS at baseline, *n* (%)	23 (31.5)	20 (29.0)	22 (30.1)	10 (28.6)	10 (28.6)
COPD severity, *n* (%)					
Moderate	53 (72.6)	47 (68.1)	51 (69.9)	20 (57.1)	20 (57.1)
Severe	20 (27.4)	22 (31.9)	22 (30.1)	14 (40.0)	14 (40.0)
Very severe	0	0	0	1 (2.9)	1 (2.9)
Mean COPD duration, years (SD)	6.8 (5.9)	7.2 (6.1)	7.1 (6.0)	6.3 (4.7)	6.3 (4.7)
Mean pre-bronchodilator FEV_1_					
mL (SD)	1410 (461)	1396 (466)	1414 (460)	1406 (542)	1406 (542)
% predicted (SD)	52.54 (13.97)	51.70 (14.20)	52.33 (14.11)	48.34 (16.20)	48.34 (16.20)
Mean post-bronchodilator FEV_1_					
mL (SD)	1542 (435)	1521 (434)	1546 (434)	1525 (538)	1525 (538)
% predicted (SD)	57.69 (13.50)	56.61 (13.61)	57.44 (13.68)	52.51 (15.35)	52.51 (15.35)
Baseline IC^a^, mL (SD)	1877 (527)	1913 (560)	1925 (546)	1979 (656)	1942 (632)
Average daily rescue medication use at baseline^b^, puffs/day (SD)	2.5 (3.5)	2.6 (3.7)	2.6 (3.6)	3.4 (3.4)	3.4 (3.4)
Patients with a moderate or severe COPD exacerbation^c^ within the past 12 months^d^, *n* (%)	13 (17.3)	12 (16.7)	12 (16.4)	10 (25.0)	10 (25.0)
Patients hospitalized/ER room treatment within the past 12 months^d^, *n* (%)	4 (5.3)	5 (6.9)	5 (6.8)	0	0

*COPD* chronic obstructive pulmonary disease, *FEV*_*1*_ forced expiratory volume in 1 s, *GFF* glycopyrrolate/formoterol fumarate, *IC* inspiratory capacity, *ICS* inhaled corticosteroids, *MDI* metered dose inhaler, *mITT* modified intent-to-treat, *SD* standard deviation, *SMI* Soft Mist™ inhaler, *TIO* open-label tiotropium

^a^Baseline IC was defined as the mean of the pre-dose values on the first day of each treatment period, where the mean of the 30- and 60-min values for each visit day was obtained, and then the visit means were averaged

^b^Baseline rescue medication use was defined as the average daily number of puffs used over the 7 days prior to the date of first dose in Treatment Period 1

^c^A COPD exacerbation was defined as a change in the subject’s baseline dyspnea, cough, and/or sputum (increase in volume or change in color towards purulence) that lasted ≥3 days, was beyond normal day to day variations, was acute in onset, and may have warranted a change in regular medication

^d^Safety population. PT003011: GFF MDI, *n* = 75; Placebo MDI, *n* = 72; TIO SMI, *n* = 73. PT003012: GFF MDI, *n* = 40; Placebo MDI, *n* = 40

### Efficacy

**Fig. 2:**
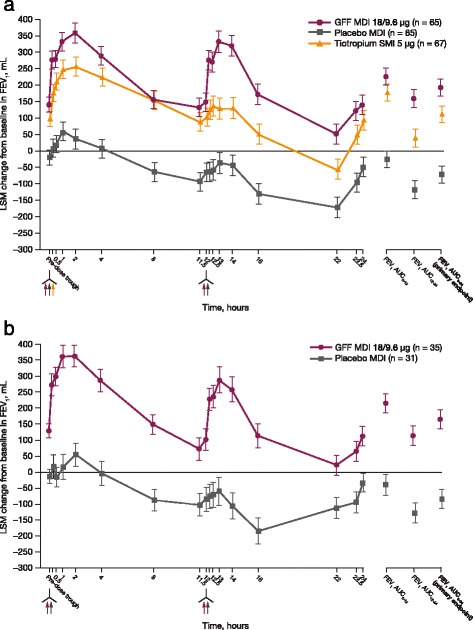
Adjusted change from baseline in FEV_1_ over 24 h on day 29. Data shown are ± SE for the mITT population in (**a**) PT003011 and (**b**) PT003012. AUC, area under the curve; FEV_1_, forced expiratory volume in 1 s; GFF, glycopyrrolate/formoterol fumarate; LSM, least squares means; MDI, metered dose inhaler; mITT, modified intent-to-treat; SE, standard error; SMI, Soft Mist™ inhaler

In both studies, treatment with GFF MDI led to significant improvements in the primary efficacy endpoint, change from baseline in FEV_1_ AUC_0–24_ on day 29. GFF MDI treatment resulted in improvements of 265 mL and 249 mL (*p* < 0.0001 for both) for FEV_1_ AUC_0–24_ relative to placebo MDI treatment (PT003011 and PT003012, respectively; Fig. 2). In study PT003011, GFF MDI also led to an 80 mL improvement relative to open-label tiotropium SMI (*p* = 0.0001). An increase of FEV_1_ AUC relative to open-label tiotropium SMI was seen over both the 0–12 h and 12–24 h intervals. However, the magnitude of this increase was greater over the 12–24-h period. Differences between GFF MDI and open-label tiotropium SMI groups in change from baseline in FEV_1_ AUC were 48 mL (*p* = 0.0325) and 120 mL (*p* < 0.0001), respectively, for 0–12 h and 12–24 h. For GFF MDI versus placebo MDI, the improvement was similar across both time periods in both studies (Table 2).

**Table 2:** Secondary endpoints: lung-function measurements (mITT population)

	PT003011			PT003012	
	GFF MDI 18/9.6 μg	Placebo MDI	TIO SMI 5 μg	GFF MDI 18/9.6 μg	Placebo MDI
	*n* = 65	*n* = 65	*n* = 67	*n* = 35	*n* = 31
FEV_1_ AUC_12–24_ (mL) on day 29, LSM (SE)	159 (27.7)	−118 (27.7)	39 (27.5)	115 (29.9)	−127 (32.0)
*Treatment difference GFF MDI* vs *comparator LSM (95% CI)*	NA	277 (232–321)	120 (76–164)	NA	242 (165–319)
	*n* = 67	*n* = 66	*n* = 68	*n* = 35	*n* = 31
FEV_1_ AUC_0–12_ (mL) on day 29, LSM (SE)	226 (25.7)	−26 (25.8)	178 (25.6)	216 (29.8)	−39 (31.8)
*Treatment difference GFF MDI* vs *comparator LSM (95% CI)*	NA	251 (207–296)	48 (4–92)	NA	255 (182–329)
	*n* = 65	*n* = 65	*n* = 67	*n* = 35	*n* = 31
Peak change from baseline in FEV_1_ (mL) following evening dose on day 29, LSM (SE)	395 (30.9)	58 (31)	230 (30.7)	344 (34.2)	50 (36.6)
*Treatment difference GFF MDI* vs *comparator LSM (95% CI)*	NA	337 (282–392)	165 (110–219)	NA	293 (204–382)
	*n* = 67	*n* = 67	*n* = 68	*n* = 35	*n* = 31
Peak change from baseline in FEV_1_ (mL) following morning dose on day 29, LSM (SE)	406 (28.3)	129 (28.3)	325 (28.1)	410 (35.8)	134 (37.6)
*Treatment difference GFF MDI* vs *comparator LSM (95% CI)*	NA	278 (225–330)	81 (29–133)	NA	276 (206–347)
	*n* = 67	*n* = 66	*n* = 68	*n* = 35	*n* = 32
Change from baseline in morning pre-dose trough FEV_1_ on day 29 (mL), LSM (SE)	140 (22.7)	−20 (22.9)	97 (22.5)	130 (21.7)	−12 (22.7)
*Treatment difference GFF MDI* vs *comparator LSM (95% CI)*	NA	161 (106–215)	43 (−11–97)	NA	142 (90–194)
	*n* = 66	*n* = 66	*n* = 66	*n* = 35	*n* = 31
Change from baseline in morning pre-dose trough FEV_1_ on day 30 (mL), LSM (SE)	129 (28.4)	−73 (28.4)	72 (28.3)	90 (28.8)	−64 (30.2)
*Treatment difference GFF MDI* vs *comparator LSM (95% CI)*	NA	203 (153–252)	58 (8–108)	NA	154 (97–211)

*AUC* area under the curve, *CI* confidence interval, *FEV*_*1*_ forced expiratory volume in 1 s, *GFF* glycopyrrolate/formoterol fumarate, *LSM* least squares means, *MDI* metered dose inhaler; *mITT*, modified intent-to-treat, *NA* not applicable, *SE* standard error, *SMI* Soft Mist™ inhaler, *TIO* open-label tiotropium

In both studies, treatment with GFF MDI resulted in a significant difference in peak change from baseline in FEV_1_ versus placebo MDI, with numerically greater differences in the evening compared to in the morning (Table 2). Additionally, in PT003011, treatment with GFF MDI led to significant improvements in peak change from baseline in FEV_1_ versus tiotropium (81 mL in the morning and 165 mL in the evening of day 29, *p* ≤ 0.0026).

**Fig. 3:**
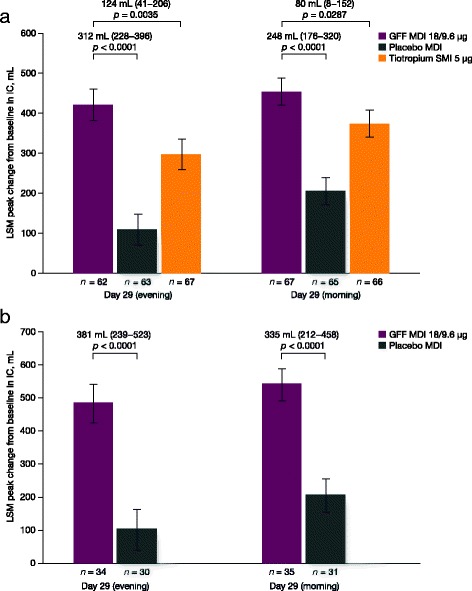
Peak change from baseline in IC on day 29 (evening/morning). Data shown are ± SE for the mITT population in (**a**) PT003011 and (**b**) PT003012. GFF, glycopyrrolate/formoterol fumarate; IC, inspiratory capacity; LSM, least squares means; MDI, metered dose inhaler; mITT, modified intent-to-treat; SE, standard error; SMI, Soft Mist™ inhaler

GFF MDI treatment improved morning pre-dose trough FEV_1_ versus placebo MDI in both studies, and versus open-label tiotropium SMI in PT003011. For all parameters, improvements were greater on day 30 than on day 29 (Table 2). Improvements in peak change from baseline in inspiratory capacity with GFF MDI compared to placebo MDI or open-label tiotropium SMI followed a similar trend as peak change from baseline in FEV_1_ and were greater following the evening dose compared to the morning dose (Fig. 3). After both doses, differences between the GFF MDI and open-label tiotropium SMI groups on day 29 were significant, with a 124 mL (*p* = 0.0035) difference between the two treatment groups in the evening, and an 80 mL (*p* = 0.0287) difference in the morning (Fig. 3a).

**Fig. 4:**
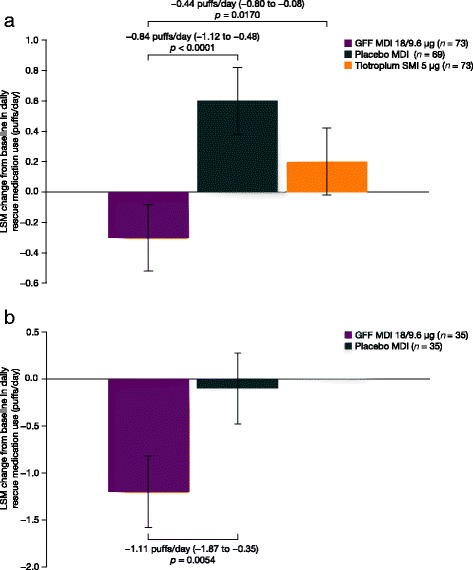
Change from baseline in average daily rescue medication use over 4-week treatment period. Data shown are ± SE for mITT population in (**a**) PT003011 and (**b**) PT003012. GFF, glycopyrrolate/formoterol fumarate; LSM, least squares means; MDI, metered dose inhaler; mITT, modified intent-to-treat; SE, standard error; SMI, Soft Mist™ inhaler

As well as resulting in significant improvements in spirometry measurements, patients treated with GFF MDI used significantly less rescue medication (−0.84 [*p* < 0.0001] and −1.11 [*p* = 0.0054] puffs/day in PT003011 and PT003012, respectively) than those treated with placebo MDI (Fig. 4). For GFF MDI versus open-label tiotropium SMI, rescue medication use was reduced by 0.44 puffs/day (*p* = 0.017).

#### Post-hoc analysis of pooled data

In a post-hoc analysis of pooled data from both studies, 72.1% of patients treated with GFF MDI achieved a >15% increase from baseline in inspiratory capacity on day 29 in the evening, compared to 19.0% of patients treated with placebo MDI and 47.0% of patients treated with open-label tiotropium SMI (Table 3). Inspiratory capacity responder analyses with thresholds of >10%, >20%, >200 mL, >300 mL, and >400 mL on day 29 demonstrated that consistently higher proportions of patients treated with GFF MDI achieved a response compared to patients treated with placebo MDI or open-label tiotropium SMI in both the morning and evening assessments (Table 3).

**Table 3:** Pooled analysis of IC response on day 29 (mITT population)

	Response in peak IC			Treatment versus comparator, OR (95% CI)		
	GFF MDI 18/9.6 μg *n* = 102	Placebo MDI *n* = 99	TIO SMI 5 μg *n* = 68	GFF MDI vs placebo MDI	GFF MDI vs TIO SMI	TIO SMI vs placebo MDI
Patients achieving a response (evening), %						
> 10%	83.5	31.5	71.1	11.00 (4.12–29.43)	2.05 (0.83–5.10)	5.36 (2.13–13.49)
> 15%	72.1	19.0	47.0	10.98 (4.34–27.79)	2.91 (1.27–6.68)	3.77 (1.58–8.98)
> 20%	54.8	15.3	31.4	6.70 (2.98–15.08)	2.65 (1.27–5.51)	2.53 (1.12–5.74)
> 200 mL	83.1	27.8	61.3	12.78 (4.70–34.79)	3.12 (1.25–7.79)	4.10 (1.68–9.99)
> 300 mL	66.3	20.0	43.4	7.84 (3.47–17.74)	2.56 (1.22–5.37)	3.06 (1.38–6.79)
> 400 mL	46.5	10.3	20.9	7.58 (3.09–18.63)	3.29 (1.42–7.61)	2.31 (0.89–5.96)
Patients achieving a response (morning), %						
> 10%	92.1	47.2	75.2	12.94 (4.57–36.68)	3.82 (1.38–10.57)	3.39 (1.39–8.30)
> 15%	81.4	30.1	65.5	10.16 (4.11–25.14)	2.31 (0.98–5.46)	4.39 (1.85–10.44)
> 20%	75.6	12.5	46.8	21.72 (6.58–71.65)	3.53 (1.32–9.47)	6.15 (2.06–18.35)
> 200 mL	89.5	40.7	72.8	12.37 (4.82–31.78)	3.17 (1.27–7.94)	3.90 (1.70–8.95)
> 300 mL	79.1	23.6	67.4	12.24 (4.62–32.47)	1.83 (0.75–4.45)	6.69 (2.51–17.80)
> 400 mL	67.9	13.3	34.0	13.84 (5.24–36.54)	4.12 (1.77–9.60)	3.36 (1.33–8.51)

*CI* confidence interval, *GFF* glycopyrrolate/formoterol fumarate, *IC* inspiratory capacity, *MDI* metered dose inhaler, *mITT* modified intent-to-treat, *OR* odds ratio, *SMI* Soft Mist™ inhaler, *TIO* open-label tiotropium

### Safety and tolerability

GFF MDI was generally well tolerated in both studies, although in PT003011 there was a higher incidence of treatment-emergent AEs in the GFF MDI treatment group. The most common AEs are summarized in Table 4. Serious AEs occurred at a similar rate in the GFF MDI treatment groups as in the placebo MDI groups. In PT003011, one serious AE of COPD in the GFF MDI group was considered related to study drug by the investigator. No deaths occurred during either of these studies.

**Table 4:** Summary of adverse events^a^ (safety population)

	PT003011			PT003012	
	GFF MDI 18/9.6 μg (*n* = 75)	Placebo MDI (*n* = 72)	TIO SMI 5 μg (*n* = 73)	GFF MDI 18/9.6 μg (*n* = 40)	Placebo MDI (*n* = 40)
Patients with ≥1 TEAE	19 (25.3)	15 (20.8)	16 (21.9)	7 (17.5)	10 (25.0)
Patients with ≥1 treatment-related TEAE	7 (9.3)	4 (5.6)	3 (4.1)	0	1 (2.5)
Patients with ≥1 serious TEAE	2 (2.7)	2 (2.8)	2 (2.7)	1 (2.5)	1 (2.5)
Patients with TEAEs leading to study discontinuation	3 (4.0)	1 (1.4)	1 (1.4)	1 (2.5)	1 (2.5)
Adverse events occurring in ≥2 patients in any group					
Respiratory, thoracic, and mediastinal disorders	2 (2.7)	4 (5.6)	3 (4.1)	0	5 (12.5)
Dyspnea	0	0	1 (1.4)	0	2 (5.0)
Sinus congestion	0	0	0	0	2 (5.0)
Cough	1 (1.3)	2 (2.8)	1 (1.4)	0	1 (2.5)
Infections and infestations	6 (8.0)	2 (2.8)	4 (5.5)	3 (7.5)	2 (5.0)
Furuncle	0	0	2 (2.7)	0	0
Vascular disorders	0	0	0	1 (2.5)	3 (7.5)
Hypertension	0	0	0	1 (2.5)	2 (5.0)
Gastrointestinal disorders	5 (6.7)	4 (5.6)	5 (6.8)	0	0
Constipation	2 (2.7)	1 (1.4)	0	0	0
Vomiting	0	0	3 (4.1)	0	0
Nervous system disorders	4 (5.3)	2 (2.8)	2 (2.7)	0	0
Headache	1 (1.3)	2 (2.8)	1 (1.4)	0	0
Tremor	3 (4.0)	0	0	0	0
Musculoskeletal and connective tissue disorders	2 (2.7)	2 (2.8)	0	0	0
Back pain	1 (1.3)	2 (2.8)	0	0	0

*GFF* glycopyrrolate/formoterol fumarate, *MDI* metered dose inhaler, *SMI* Soft Mist™ inhaler, *TEAE* treatment-emergent adverse event, *TIO* open-label tiotropium

^a^Data shown are number of patients (%)

## Discussion

In these two phase IIIb studies (PT003011 and PT003012), the LAMA/LABA FDC GFF MDI 18/9.6 μg BID formulated using innovative co-suspension delivery technology improved lung-function parameters in patients with moderate-to-very severe COPD over 24 h. GFF MDI is the first available LAMA/LABA FDC maintenance treatment for COPD patients to be delivered using a MDI. The use of a MDI could be particularly beneficial in patients with hyperinflation and reduced inspiratory capacity, who may find it difficult to achieve the minimum inspiratory flow required for a dry powder inhaler [13, 14].

The results of PT003011 and PT003012 are in agreement with the PINNACLE-1 and -2 studies, which showed significant improvements in lung-function measures such as morning pre-dose trough FEV_1_ over 24 weeks of treatment with GFF MDI, compared with placebo MDI [9]. Together with the PINNACLE-3 study [11], the data presented here provide additional evidence for both long- and short-term efficacy and safety of GFF MDI as a maintenance treatment for moderate-to-very severe COPD.

In previous studies assessing 24-h lung-function profiles of LAMA/LABA combinations, patients treated with tiotropium/olodaterol (QD via a SMI) or umeclidinium/vilanterol (QD via a dry powder inhaler) also showed significant improvements in lung function over a 24-h period compared with patients receiving placebo or monocomponents [15, 16]. However, unlike in our study with a BID dosing regimen, in these QD dosing studies improvements were lower in the 12–24-h period than in the 0–12-h period [15, 16]. Few studies have assessed the 24-h lung-function profile of a bronchodilator with a BID dosing regimen [17].

A previous study investigating the effect of adding formoterol QD or BID to tiotropium QD treatment found that the addition of formoterol BID led to significant improvements in FEV_1_ over 12–24 h compared to tiotropium alone [18]. In another study comparing formoterol BID to olodaterol QD, the evening dose of formoterol led to an increase in night-time FEV_1_ compared to placebo and olodaterol [19], suggesting that BID dosing of a LABA can also provide overnight lung-function benefits in patients with COPD. In both PT003011 and PT003012, patients treated with GFF MDI BID showed significantly greater changes from baseline in spirometry measures over the whole 24-h period compared with patients receiving placebo MDI or open-label tiotropium SMI QD. These improvements were most noticeable during the 12–24-h period of the study, suggesting that GFF MDI BID treatment could provide prolonged lung-function benefits in the second half of the day (including overnight) compared to once-daily LAMA and possibly LAMA/LABA treatment.

Circadian variations in FEV_1_ have been reported in patients with COPD, with peak values observed during daytime hours and a decrease in FEV_1_ occurring overnight [20, 21]. In a study investigating the effect of the timing of tiotropium dosing on overnight FEV_1_, administration of tiotropium in the evening did not significantly improve overnight FEV_1_ in comparison to dosing in the morning [21]. In the present study, the administration of GFF MDI BID did improve overnight FEV_1_ in comparison to placebo MDI BID and open-label tiotropium SMI QD (Table 2; Fig. 2).

GFF MDI BID also provided significant improvements in inspiratory capacity, which were sustained over a 24-h period. An additional post-hoc analysis of data pooled from PT003011 and PT003012 found that patients receiving GFF MDI BID were more likely to achieve an inspiratory capacity response than those treated with placebo MDI BID and open-label tiotropium SMI QD, which was consistent over a range of response thresholds and for both the morning and evening assessments.

It has been demonstrated previously that low inspiratory capacity is correlated with decreased exercise tolerance [22] and increased dyspnea in patients with COPD [23–25], although one study found that dyspnea was more closely linked to improvements in FEV_1_ than inspiratory capacity [26]. In addition, inspiratory capacity has been shown to be a predictor of all-cause and respiratory mortality in patients with COPD; and is linked to hospitalizations due to COPD exacerbations [27]. Hence, improving inspiratory capacity may improve exercise capacity and dyspnea symptoms, and have an impact on long-term disease outcomes.

A potential limitation of this study was the open-label nature of the tiotropium arm, whereas GFF MDI and placebo MDI were supplied blinded. However, this was in part mitigated by dosing patients in the clinic for the 24-h assessment at the end of each treatment period. A strength of this study was the crossover design, which provided equivalent power to a much larger parallel study. This allowed for within-subject comparison of active treatment versus placebo, which provides a better correction for diurnal variation than a parallel study design.

## Conclusions

These studies showed that, in patients with moderate-to-very severe COPD, the twice-daily LAMA/LABA treatment GFF MDI resulted in a reduction in airflow limitation and hyperinflation, demonstrated by significant benefits on inspiratory capacity in comparison to placebo MDI and once-daily open-label tiotropium SMI. These effects were sustained over the whole 24-h period. The studies also evidenced a similar safety and tolerability profile for GFF MDI, placebo MDI, and open-label tiotropium SMI. Benefits compared to once-daily tiotropium SMI were greater in the 12–24-h period, suggesting that GFF MDI twice-daily treatment could offer longer-lasting improvements in night-time lung function over once-daily dosing regimens.

## Abbreviations

AE: Adverse event
AUC_x-xx_: Area under the curve from X to XX hours
BID: Twice daily
CI: Confidence interval
COPD: Chronic obstructive pulmonary disease
FDC: Fixed-dose combination
FEV_1_: Forced expiratory volume in 1 s
GFF: Glycopyrrolate/formoterol fumarate
IC: Inspiratory capacity
ICS: Inhaled corticosteroids
LABA: Long-acting β_2_-agonist
LAMA: Long-acting muscarinic antagonist
LSM: Least squares mean
MDI: Metered dose inhaler
mITT: modified intent-to-treat
NA: Not applicable
QD: Once daily
SD: Standard deviation
SE: Standard error
SMI: Soft Mist™ Inhaler
TEAE: Treatment-emergent adverse event
TIO: Open-label tiotropium
